# Circadian rhythms are associated with higher amyloid-β and tau and poorer cognition in older adults

**DOI:** 10.1093/braincomms/fcaf322

**Published:** 2025-09-08

**Authors:** Joanna L Eckhardt, Lisette Isenberg, Vahan Aslanyan, Teresa Monreal, Joy Stradford, Laura Fenton, Joey A Contreras, Wendy J Mack, Judy Pa

**Affiliations:** Alzheimer’s Disease Cooperative Study (ADCS), Department of Neurosciences, University of California, San Diego, La Jolla, CA 92093, USA; Alzheimer’s Disease Cooperative Study (ADCS), Department of Neurosciences, University of California, San Diego, La Jolla, CA 92093, USA; Department of Population and Public Health Sciences, Keck School of Medicine, University of Southern California, Los Angeles, CA 90007, USA; Mark and Mary Stevens Neuroimaging and Informatics Institute, Keck School of Medicine, University of Southern California, Los Angeles, CA 90007, USA; SDSU/UCSD Joint Doctoral Program in Clinical Psychology, University of California, San Diego, La Jolla, CA 92093, USA; Department of Psychology, University of Southern California, Los Angeles, CA 90007, USA; Alzheimer’s Disease Cooperative Study (ADCS), Department of Neurosciences, University of California, San Diego, La Jolla, CA 92093, USA; Department of Population and Public Health Sciences, Keck School of Medicine, University of Southern California, Los Angeles, CA 90007, USA; Alzheimer’s Disease Cooperative Study (ADCS), Department of Neurosciences, University of California, San Diego, La Jolla, CA 92093, USA

**Keywords:** circadian rhythms, Alzheimer’s disease, accelerometry, pathology, cognition

## Abstract

Several studies implicate circadian rhythm disturbances in Alzheimer’s disease. However, very little is known about how circadian rhythms are associated with Alzheimer’s pathological biomarkers in older adults at early stages of the disease, and how these relationships map onto cognition. This cross-sectional study used 24-h accelerometry data to investigate the relationships between circadian rhythms, amyloid-β (Aβ), tau, and cognition in 68 older adults with objective early cognitive impairment. Participants wore GENEActiv accelerometers for ∼1 month (mean = 31.8 days). Circadian rhythms measures were quantified from accelerometer data and included acrotime (average time of day of peak activity) and intradaily variability (IV) (average circadian rhythm fragmentation within a day). Aβ was measured as a composite, and tau (*n* = 67) was measured in Braak staging regions of interest I/II and III/IV using positron emission tomography. The cognitive domains used were verbal memory (California Verbal Learning Test short delay free recall) and attention/processing speed (Digit Symbol Substitution Test). Multivariable linear regression models were conducted to test for associations between circadian rhythms and the outcome variables of Aβ, tau, and cognition. The moderating effects of age, sex, and *apolipoprotein E4* (*APOE4*) carrier status were assessed in these associations. To investigate mechanistic pathways through which circadian rhythms may impact cognition, exploratory mediation analyses were conducted *post hoc*. Models were adjusted for age, sex, *APOE4* carrier status, and years of education. The study included 68 older adults (mean age = 66.8 years, age range = 55–80 years, 63.2% female, 26.5% *APOE4* carriers). Earlier acrotime was associated with higher Aβ and tau, the former of which was stronger in *APOE4* carriers relative to non-carriers. Higher IV was related to higher tau in Braak regions III/IV. Age and sex modified the association between IV and tau, in which the relationships strengthened with increasing age and disproportionately affected men. Earlier acrotime was associated with worse verbal memory, but later acrotime was associated with worse attention/processing speed. Tau in Braak regions I–IV mediated the relationship between acrotime and verbal memory. The insights from this study revealed that circadian rhythms were associated with Aβ, tau, and cognition in older adults with objective early cognitive impairment. We provide novel evidence for tau as a biological mediator in the relationship between circadian timing and cognition. This work identified circadian rhythms as a promising point of intervention to reduce Alzheimer’s disease risk and potentially mitigate pathological progression and cognitive decline.

## Teaser

Eckhardt *et al*. report that variable circadian rhythm timing and strength were related to higher levels of Alzheimer’s proteins, Aβ, and tau, as well as lower cognition in older adults. These relationships disproportionately affected men, *APOE4* carriers, and those of older age. Tau levels mediated the association between timing and verbal memory.

## Graphical Abstract

**Graphical Abstract:**
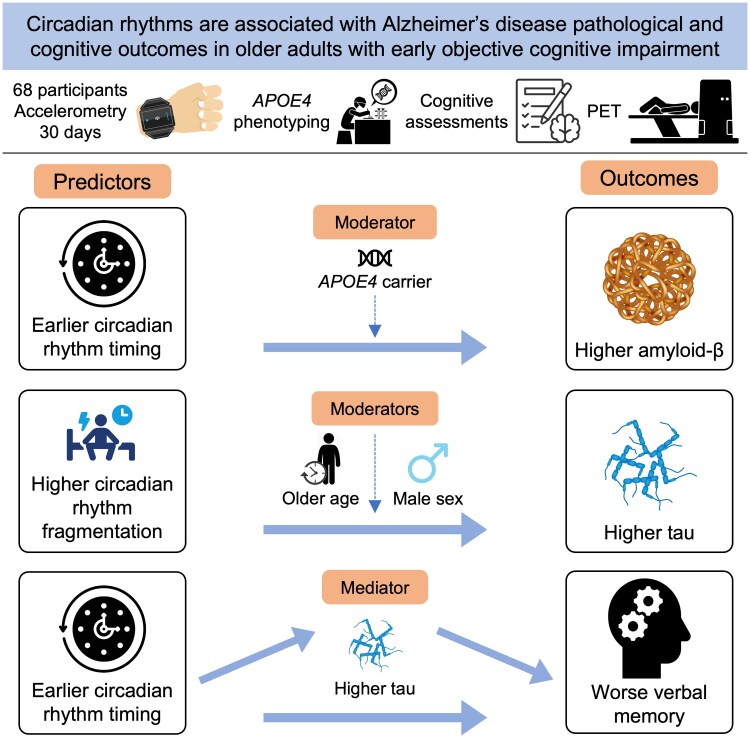

## Introduction

Circadian rhythms are evolutionarily conserved adaptations of physiological, mental, and behavioural changes entrained to the 24-h light–dark cycle of Earth’s rotation. These rhythms are primarily entrained by light signalling, which in turn regulates the release of melatonin to promote drowsiness and sleep onset,^1,2^ as well as cortisol to initiate an awakening response,^3^ among several other hormones. Circadian rhythms exert influence over many systems that are affected in Alzheimer’s disease, and numerous studies identify circadian changes across disease progression. For example, reduced levels of melatonin and melatonin receptor expression,^4-10^ as well as elevated cortisol,^11^ have been reported in mild cognitive impairment (MCI) and Alzheimer’s populations. Moreover, disrupted circadian rhythms are widely observed in Alzheimer’s patients. These include fragmented sleep–wake cycles,^12-15^ reduced day-to-day rhythm stability,^12-14,16,17^ decreased rhythm amplitude,^13,14,18^ delayed circadian phase,^13,17-19^ irregular body temperature rhythms,^13,17,19-21^ and sundowning,^17,20,22^ which is the chronobiological worsening of neuropsychiatric and behavioural symptoms in the late afternoon and evening.

Despite the abundant research linking symptoms of disrupted circadian rhythms and Alzheimer’s disease, their role as a modifiable risk factor for preventing MCI and Alzheimer’s disease remains poorly understood. Evidence from studies investigating circadian rhythms in early Alzheimer’s stages supports the idea that circadian dysfunction may occur before the onset of overt cognitive symptoms,^23-30^ suggesting that circadian changes may be an early risk factor for developing MCI and dementia. Despite these findings, very little is known about how circadian rhythm timing and fragmentation are associated with the pathological biomarkers of Alzheimer’s disease, amyloid-β (Aβ) and tau, in older adults, and how these relationships map onto cognition.

The present study sought to address this gap in the literature using data from wrist-worn accelerometers, which provide continuous, non-invasive, objective, high-resolution quantification of 24-h activity data. Cross-sectional baseline accelerometry data from the Lifestyle Enriching Activities for Research in Neuroscience Intervention Trial were examined in 68 sedentary older adults with objective early cognitive impairment, defined as performance on cognitive testing measuring at least 1 SD below the age-matched normative values. These participants represent a cognitively vulnerable population with subtle, subclinical cognitive decline that may precede clinical MCI. Circadian rhythm timing was assessed with acrotime, a common measure for the average time of day of peak activity. Circadian fragmentation was measured with intradaily variability (IV) (the average circadian rhythm fragmentation within a day). We investigated the relationships between circadian rhythms and the outcome measures of Aβ PET, tau PET, and cognition (verbal memory and attention/processing speed). We also explored how these relationships varied by age, sex, and *apolipoprotein E4* (*APOE4*) carrier status. Mediation models were used to investigate Aβ and tau pathology as mechanistic mediators through which circadian rhythms may impact cognition. We hypothesized that higher circadian fragmentation and variable circadian timing would be associated with higher Aβ and tau burden, as well as lower cognitive scores. We further hypothesized that pathology levels would mediate the relationships between circadian rhythms and cognition.

## Materials and methods

### Study participant details

Cross-sectional baseline data from the Lifestyle Enriching Activities for Research in Neuroscience Intervention Trial (LEARNit), collected between August 2016 and July 2022, were used for this study (https://www.clinicaltrials.gov/study/NCT02726906). The current study sample included 68 sedentary older adults (ages 55–80) with objective early cognitive impairment, characterized by screening performance measuring at least 1 SD below the age-matched normative values on tests of attention, executive function, or memory. Participants did not have a clinical diagnosis of cognitive impairment. At baseline, participants completed tests of cognition, Aβ and tau PET, and *APOE4* phenotyping. All participants who completed baseline actigraphy, brain imaging, and cognitive testing prior to randomization were included in the study (*n* = 68). Participants were recruited from the Greater Los Angeles Area and had no current or prior history of any major psychiatric illness, organ failure, epilepsy, or hydrocephalus. LEARNit participant characteristics are located in Table 1.

**Table 1:** Participant characteristics of study sample

Characteristic	*n* = 68
Age at accelerometry collection (mean, SD)	66.8 (6.6)
Women (%)	63.2
*APOE4* carrier status (%)	26.5
Years of education (mean, SD)	16.8 (2.5)
Montreal Cognitive Assessment Score (mean, SD)	26.1 (2.7)
Ethnicity, Hispanic/Latino (%)	13.2
Race (%)	
White	76.5
African American	13.2
Asian	4.4
American Indian or Alaska Native	1.5
Other	4.4
More than one race	4.4
Circadian rhythms (mean, SD)	
IV	0.91 (0.20)
Acrotime (hour of day)	14.26 (1.53)
Aβ PET composite SUVR (mean, SD)	1.00 (0.17)
Tau PET SUVR (*n* = 67) (mean, SD)	
Braak ROIs I/II	1.20 (0.13)
Braak ROIs III/IV	1.15 (0.098)
Cognition (mean, SD)	
CVLT short delay free recall total score	10.4 (3.4)
WAIS Digit Symbol Substitution score	60.9 (15.1)

See also Supplementary Table 1.

### Actigraphy

#### Processing of raw accelerometer data

All processing of accelerometer data was conducted in R version 4.3.1 (June 2023).^31^ Participants wore their GENEActiv (Activinsights Ltd)^32^ accelerometers for ∼1 month (average: 31.8 ± 4.2 days, range: 21–45 days) prior to brain imaging and cognitive testing. Initial processing of raw data was conducted with GGIR: Raw Accelerometer Data Analysis (version 3.0-1).^33,34^ The summary measure of dynamic acceleration produced by GGIR is the Euclidean Norm Minus One (ENMO)^35^ at a sampling rate of 1/5 Hz (one ENMO value recorded every 5 s).

#### Adjusting for daylight saving time

For the participants whose accelerometer collection periods spanned daylight saving time starting or ending (*n* = 15), 1 week of accelerometry data was removed starting on the day of their time change.^36^

#### Circadian rhythm variables from accelerometry

The two circadian rhythm measures included in this study are acrotime and IV. Acrotime is extracted from Extended Cosinor Analysis,^37-39^ and IV is derived using non-parametric methods.^40^

The R package ActCR: Extract Circadian Rhythms Metrics from Actigraphy Data (version 0.3.0)^41,42^ was used to extract acrophase, which measures the average time over 24 h of high values recurring in each cycle, measured in units of radians. Acrotime is the acrophase measured in hours (military time). This measure is considered the average time of day of peak activity. A lower acrotime is indicative of an earlier average peak activity time, whereas a higher acrotime is indicative of a later average peak activity time.

The R package nparACT: Non-Parametric Measures of Actigraphy (version 0.8)^43,44^ was used to extract IV, which has been widely used in several studies as a measure of circadian rhythm fragmentation and sleep–wake cycle disturbance.^12,16,45-48^ IV quantifies transitions between rest-activity periods. Low IV is characterized by long stretches of activity in daytime and rest in nighttime. High IV consists of rhythms that fluctuate between low and high activity within a day and may signify the presence of daytime napping and/or nighttime activity. IV is measured on a scale of 0–2, where zero is indicative of a nearly perfect sinusoidal wave and two is Gaussian noise.^47^

#### Preparing accelerometry data for circadian rhythm variable extraction

GGIR-processed accelerometer data were checked for missing ENMO samples over the collection period using the R package padr: Quickly Get Datetime Data Ready for Analysis (version 0.6.2).^49^

The ActCR’s *ActExtendCosinor* function requires that the time series data are divisible by 1440 (60-s epochs over 24 h). As such, accelerometer data were downsampled with padr into averaged minute-level data (sampling rate of 1/60 Hz). *ActExtendCosinor* also requires an input vector of ENMO acceleration values with no corresponding date–time values. To correctly compute acrotime, it is thus necessary that the first ENMO value corresponds to midnight on the first full day of collection. To address this requirement, downsampled accelerometer data were sliced to start at 00:00:00 on the first full day of collection and end at 23:59:00 on the last full day of collection.

Each participant’s time series data were then plotted and manually inspected for visual abnormalities. Some participants had extra days beyond their collection period that consisted of nearly perfect sinusoidal activity. This abnormality was likely due to participant non-wear time after their ∼30-day collection period had ended, which GGIR misinterpreted as part of the collection period and then imputed. All participants’ data were checked for unnaturally sinusoidal data, and these days were removed.

Following these modifications, the *ActExtendCosinor* function was used to extract acrotime for the 68 LEARNit participants using ‘window = 1’ (each epoch was in a 1-min window), and the *nparACT_base* function was used to calculate IV using ‘SR = 1/60’ (sampling rate in Hz = one sample every 60 s).

### Imaging

Aβ PET data were available for 68 LEARNit participants, and tau PET data were available for 67 participants. Neuroimaging acquisition for the LEARNit study has been described previously.^50^

#### MRI acquisition

Structural T_1_-weighted (T1w) MRI was obtained with a Siemens 3T Prisma scanner under the following parameters: repetition time/echo time, 2400/2.2 ms; field of view, 176 × 240 × 256 mm; and resolution, 1.0 mm^3^ isotropic.

#### PET image acquisition

For Aβ PET image acquisition, participants received 8.22 ± 0.54 millicurie (mCi) of intravenous injection of the tracer Neuraceq [florbetaben F 18 (FBB)]. After 90 min post-injection of FBB, 4 × 5-min frames were acquired. For tau PET, 11.84 ± 10.75 mCi of [^18^F]flortaucipir (FTP) tracer was given to participants through intravenous injection. After 75 min post-injection of FTP, 6 × 5-min frames were acquired. All injections occurred outside the scanner room.

#### Neuroimaging data processing

Data processing for Aβ and tau consisted of an in-house PET processing pipeline that has been previously detailed^50-52^ and is summarized below.

A cohort-specific group template was created using T1w scans of all participants with MRI data available using tools from the Advanced Normalization Tools (ANTs) package.^53,54^ Specifically, the ANTs cortical thickness pipeline was utilized to create a LEARNit-study-specific group template by moving each of the participants’ T1w images from their baseline visit into the same group space. The resulting normalized single-subject T1w image was then used as a reference for the participant’s subsequent MRI/PET image registration and generation of regions of interest (ROIs). One participant with PET data available was unable to complete the MRI; therefore, an age- and gender-matched MRI was assigned to this participant and utilized as a template for subsequent PET registration. After each subject’s T1 image was normalized to the group template, it was further processed using FreeSurfer (version 6.0.0).^55^

Motion correction was performed on the dynamic FBB and FTP PET images by aligning each frame to an average image. These motion-corrected PET frames were then averaged and assessed for residual motion. Subsequently, images were co-registered to the T1w image template space using ANTs tools, and smoothed with an 8 mm Gaussian kernel.

For the FBB tracer measuring Aβ, the whole cerebellum was used as a reference region.^56^ The cerebellum or a portion of the cerebellum is generally used as the reference for Aβ and tau PET imaging because this brain area is not impacted by pathology until the late stages of Alzheimer’s disease.^57^

Bilateral grey and white matter labels in the cerebellum from FreeSurfer were combined, eroded by one voxel to reduce partial volume effects (PVE)—the loss of activity in small regions due to limited resolution—and then moved into the FBB PET space.

For the FTP tracer measuring tau, the reference region used was the inferior cerebellar grey matter with dorsal regions removed, as suggested by prior work.^56^ The dorsal regions were removed from the cerebellar ROI by performing cerebellar segmentation with a spatially unbiased atlas template of the cerebellum and brainstem (SUIT),^58-61^ and subsequently excluding dorsal regions. The dorsal cerebellar SUIT ROI was then masked by the FreeSurfer cerebellar grey matter mask, eroded by one voxel to reduce PVE, and moved into the FTP PET space. The average PET signal was then extracted from reference ROIs in native PET space for both FBB and FTP.

To generate standardized uptake value ratio (SUVR) images that quantify Aβ and tau, the PET signal in each voxel was divided by the average signal in the respective reference region. These images were corrected for PVE. To characterize levels of PET binding, composite ROIs were created for Aβ and tau. In this study, composite Aβ was used, which consists of FBB scores across the frontal, parietal, lateral temporal, and cingulate cortices.^62^ For tau as measured by FTP, ROIs were created using FreeSurfer labels that correspond to Braak staging.^56,63^ Weighted composite SUVRs were collected from two ROIs that correspond to traditional anatomical Braak staging of tau pathology: ROIs I/II (transentorhinal) and ROIs III/IV (limbic).^64^ Tau Braak ROIs I/II and III/IV were used as separate measures in the analyses for this study. Tau Braak ROIs V/VI (neocortical) were not examined in this study, as later Braak stage tau accumulation is unlikely to be observed in participants with only objective early cognitive impairment.

### Cognitive assessments

The cognitive domains used in the current study were verbal memory and attention/processing speed. Verbal memory was assessed with the California Verbal Learning Test (CVLT), Second Edition, short delay free recall total score.^65^ Attention/processing speed was measured using the Wechsler Adult Intelligence Scale (WAIS) Digit Symbol Substitution Test.^66^

### Statistical analysis

LEARNit participant data were de-identified prior to analysis to achieve blinding and reduce bias. All quantitative data were analysed as continuous variables. All statistical analyses were conducted in R version 4.3.1 (June 2023)^31^ and a 95% confidence interval (CI) for the size of each effect was used.

For each variable, participants with absolute values >3 SD were considered outliers and were excluded from analyses. No outliers were identified for IV or cognition (*n* = 68). Acrotime (*n* = 67) and both tau variables (*n*  *=* 66) had one outlier removed. Composite Aβ had two outliers removed (*n* = 66). Statistical tests were conducted with all available data, so the sample size varied slightly between models due to differences in outlier removals, and one participant was missing tau PET.

Two-tailed Pearson’s correlation analysis was conducted to test for associations between age and circadian rhythms. Two-tailed Wilcoxon rank-sum tests were performed to determine whether circadian rhythms differed by sex or *APOE4* carrier status. A *P*-value < 0.05 was used as a threshold for statistical significance.

Multivariable linear regression models were conducted to test for associations between the circadian rhythm (exposure) variables and the outcome variables of Aβ, tau, and cognition. All regression models were adjusted for potential confounders of age, sex, and *APOE4* carrier status. Models that included a cognitive measure were further adjusted for years of education. Regression diagnostics were performed through visual inspection to confirm that the assumptions of linear regression analyses were met. Multivariable linear regression *P*-values were False Discovery Rate (FDR)-corrected for multiple comparisons across all models tested within each outcome domain. Outcome domains consisted of Aβ (composite Aβ SUVR), tau (tau SUVR in Braak ROIs I/II and III/IV), and cognition (short delay free recall total score and Digit Symbol Substitution score). An FDR-corrected *P*-value < 0.05 was used as a threshold for statistical significance.

To assess the moderating effects of age, sex, and *APOE4* carrier status, interaction terms of these covariates with circadian rhythm variables were added to the above regression models. A *P*-value < 0.05 was used as a threshold for statistical significance. Interactions with *P*-value < 0.10 were reported and subsequently probed with stratified analyses.

To investigate mechanistic pathways through which circadian rhythms may impact cognition, exploratory mediation analyses were conducted *post hoc*. Specifically, we explored the potential mediating roles of Aβ and tau pathology in the associations of circadian rhythms with cognition; we investigated mediation for associations in which we noted statistically significant main effects before FDR correction. Mediation was conducted using the R package mediation: Causal Mediation Analysis (version 4.5.0).^67^ Mediation models were adjusted for age, sex, *APOE4* carrier status, and years of education. CIs in mediation models were calculated via bootstrapping with 5000 simulations. A *P*-value < 0.05 was used as a threshold for statistical significance. Mediation models with significant indirect effects were conducted again with the independent variable and mediator swapped to assess reverse causality.

## Results

### Participant characteristics

The study consisted of 68 sedentary older adult participants (mean age = 66.8 ± 6.6 years, 63.2% female, 26.5% *APOE4* carriers, mean education = 16.8 ± 2.5 years). One participant was missing tau PET data. Table 1 summarizes participant characteristics. Supplementary Table 1 summarizes averages for each variable by sex and *APOE4* carrier status.

**Figure 1:**
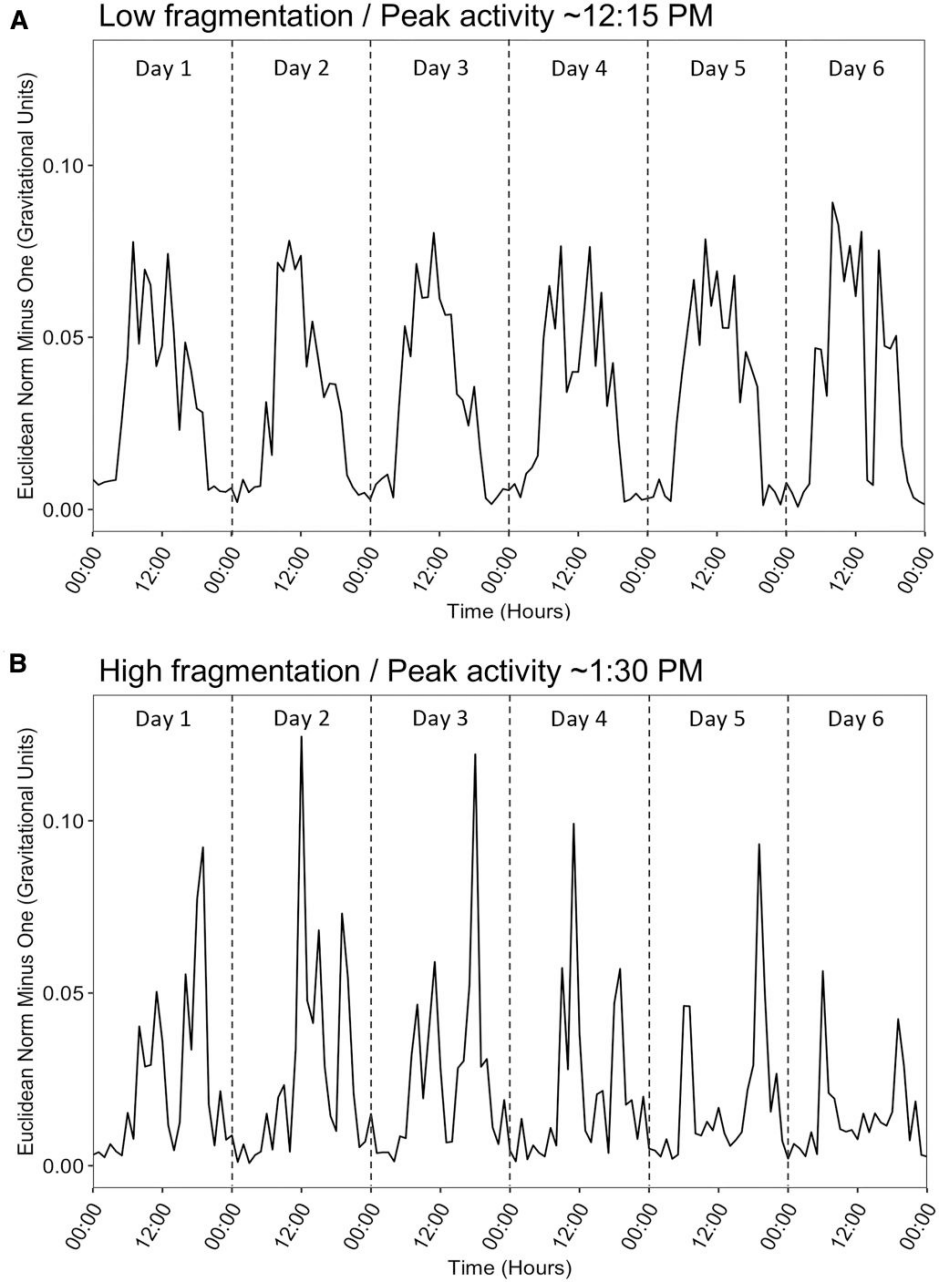
**Activity plot examples.** (**A**) A 63-year-old woman with a low IV of 0.52 and an acrotime of 12.28 (12:17 P.M.). (**B**) A 55-year-old man with a high IV of 1.16 and an acrotime of 13.62 (1:37 P.M.). Activity data for 6 days are shown for each participant. IV and acrotime are derived from the 6 days shown. 00:00 = Midnight (12 A.M.). 12:00 = Noon (12 P.M.). Vertical dashed lines separate days. Data were downsampled into averaged hourly measurements with a sampling rate of 1 ENMO (summary measure of dynamic acceleration) per hour. See also Supplementary Fig. 1.

Figure 1 demonstrates representative activity plots over 6 days from the study sample with varying magnitudes of acrotime and IV. Acrotime is demonstrated by the location of the peak of activity along the *x*-axis. Low IV in Fig. 1A is seen as a consistent sinusoidal rise of activity during daytime and a fall of activity during nighttime. High IV in Fig. 1B is demonstrated by a fragmented circadian sleep–wake cycle void of consistent rhythmicity, with fluctuations between activity and rest throughout each 24-h period. Histogram distributions for acrotime and IV are shown in Supplementary Fig. 1.

### Age is correlated with circadian timing

Age was negatively correlated with acrotime [*r* = −0.25, 95% CI: (−0.46, −0.011), *P* = 0.041, *n* = 67], suggesting that older age was associated with lower acrotime (earlier average peak activity time). IV and age were not correlated. Acrotime and IV did not differ by sex. Acrotime and IV also did not differ by *APOE4* carrier status.

### Circadian timing is associated with Aβ burden

**Figure 2:**
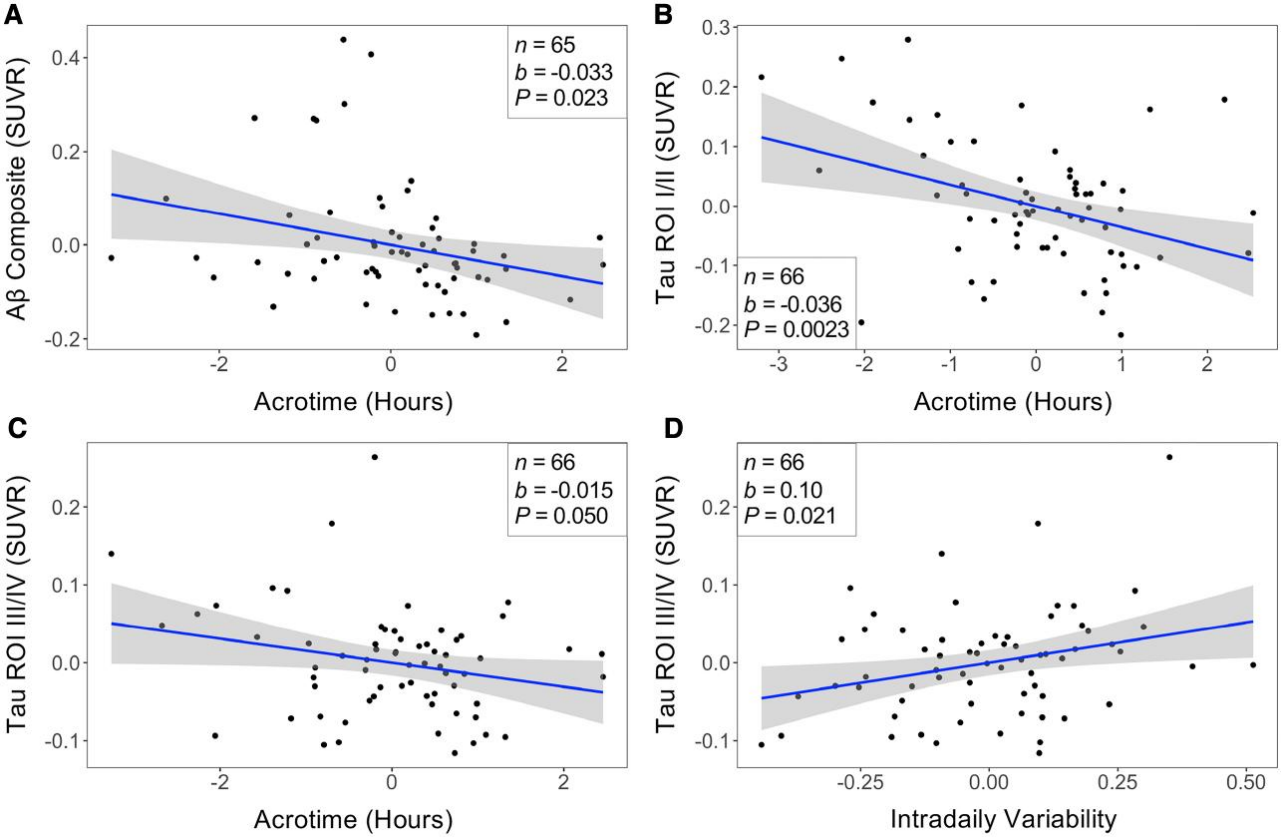
**Circadian rhythms are associated with Aβ and tau burden.** Added variable plots for the associations between (**A**) acrotime and composite Aβ, (**B**) acrotime and tau Braak ROIs I/II, (**C**) acrotime and tau Braak ROIs III/IV, and (**D**) IV and tau Braak ROIs III/IV. Each individual data point represents one participant. Lines represent the estimated change in the outcome for every unit change of the predictor. Shaded regions represent the 95% CI for these estimates. Beta coefficient (*b*) and unadjusted *P*-value (*P*) obtained from linear regression analysis adjusted for age, sex, and *APOE4* carrier status. Covariates were regressed out in the added variable plot. Acrotime is not translatable to military time due to regressing out the covariates. For reference, the mean and SD of acrotime are 14.26 (2:16 P.M.) and 1.53 (1 h and 32 min).

Acrotime was negatively associated with composite Aβ SUVR [*b* = −0.033, 95% CI: (−0.062, −0.0047), *P* = 0.023, FDR-adjusted *P* = 0.046, *n* = 65, Fig. 2A], indicating that an earlier peak activity time was associated with higher Aβ burden. IV and Aβ were not related.

### *APOE4* carrier status modifies the association between circadian timing and Aβ burden

**Figure 3:**
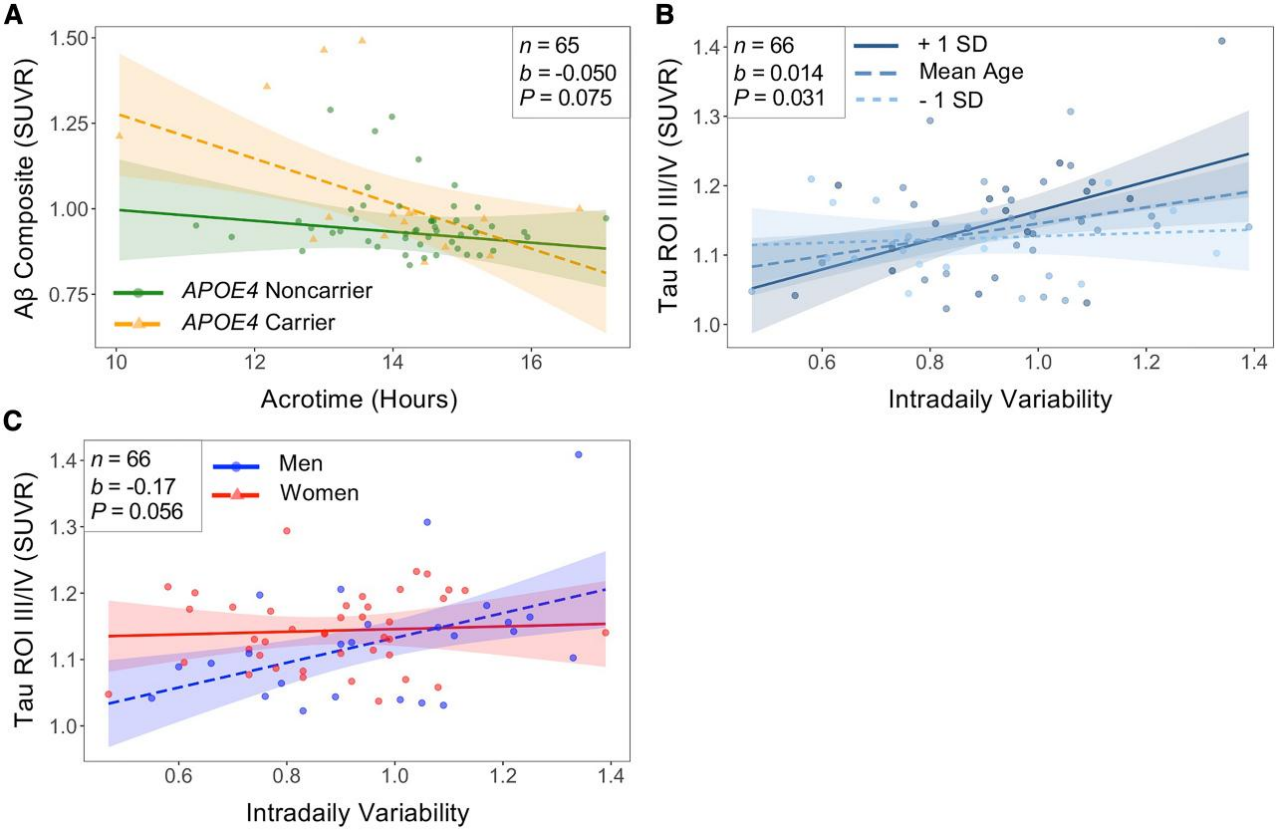
***APOE4* carrier status, age, and sex modify the associations between circadian rhythms and Aβ and tau burden.** Interaction plots for the associations between (**A**) acrotime and composite Aβ with an interaction term for *APOE4* carrier status, (**B**) IV and tau Braak ROIs III/IV with an interaction term for age, and (**C**) IV and tau Braak ROIs III/IV with an interaction term for sex. Each individual data point represents one participant. For categorical interactors shown in **A** and **C**, lines represent the estimated change in the outcome for every unit change of the predictor at specified levels of the categorical interactor (*APOE4* non-carrier versus *APOE4* carrier; men versus women). For the continuous interactor shown in **B**, the lines represent the estimated change in the outcome for every unit change of the predictor for the mean age, 1 SD above the mean age, and 1 SD below the mean age. Shaded regions represent the 95% CI for these estimates. In interaction models involving composite Aβ, beta coefficient (*b*) and *P*-value (*P*) obtained from the interaction term between acrotime and *APOE4* carrier status moderator in linear regression analysis adjusted for age, sex, and *APOE4* carrier status for a sample size of *n* = 65 (49 *APOE4* non-carriers/16 *APOE4* carriers). In interaction models involving tau, *b*, and *P* obtained from interaction terms between IV and age or sex moderator in linear regression analysis adjusted for age, sex, and *APOE4* carrier status for a sample size of *n* = 66 (25 men/41 women).

An interaction was observed between acrotime and *APOE4* carrier status in the relationship between acrotime and composite Aβ [interaction *b* = −0.050, 95% CI: (−0.10, 0.0052), *P* = 0.075, *n* = 65, Fig. 3A]. When the sample was subsequently stratified by *APOE4* carrier status, the *b-*coefficient between acrotime and composite Aβ had a higher magnitude in *APOE4* carriers [*b* = −0.055, 95% CI: (−0.15, 0.042), *P* = 0.24, *n* = 16] compared to non-carriers [*b* = −0.018, 95% CI: (−0.044, 0.0080), *P* = 0.17, *n* = 49], demonstrating that this relationship in the total sample was largely driven by *APOE4* carriers.

### Circadian timing and fragmentation are associated with tau burden

Negative associations were observed between acrotime and tau in Braak ROIs I/II [*b* = −0.036, 95% CI: (−0.059, −0.013), *P* = 0.0023, FDR-adjusted *P* = 0.0090, *n* = 66, Fig. 2B] and III/IV [*b* = −0.015, 95% CI: (−0.031, −0.0000015), *P* = 0.050, FDR-adjusted *P* = 0.067, *n* = 66, Fig. 2C]. These results indicate that an earlier peak activity time was associated with higher tau burden. IV was positively associated with tau in Braak ROIs III/IV [*b* = 0.10, 95% CI: (0.016, 0.19), *P* = 0.021, FDR-adjusted *P* = 0.041, *n* = 66, Fig. 2D], indicating that higher circadian fragmentation was significantly associated with higher tau pathology. IV and tau in Braak ROIs I/II were not associated.

### Age and sex modify the association between circadian fragmentation and tau burden in ROIs III/IV

A significant interaction was observed between IV and age in the relationship between IV and tau in Braak ROIs III/IV [interaction *b* = 0.014, 95% CI: (0.0013, 0.027), *P* = 0.031, *n* = 66, Fig. 3B]. These results indicate that the slope estimate of the association between IV and tau in Braak ROIs III/IV was 0.014 higher for each additional year increase in age. An interaction between IV and sex was also observed in the relationship between IV and tau in Braak ROIs III/IV [interaction *b* = −0.17, 95% CI: (−0.34, 0.0047), *P* = 0.056, *n* = 66, Fig. 3C]. When the sample was subsequently stratified by sex, the association between IV and tau in Braak ROIs III/IV persisted in men [*b* = 0.19, 95% CI: (0.042, 0.34), *P* = 0.014, *n* = 25], but not in women [*b* = 0.020, 95% CI: (−0.083, 0.12), *P* = 0.69, *n* = 41].

### Circadian timing is associated with verbal memory and attention/processing speed

**Figure 4:**
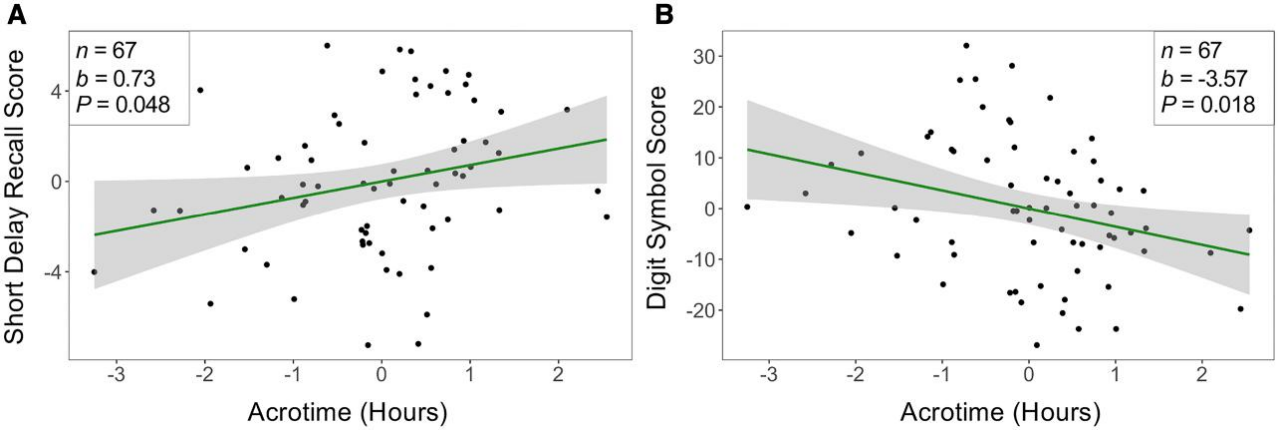
**Circadian timing is associated with verbal memory and attention/processing speed.** Added variable plots for the associations between (**A**) acrotime and short delay free recall total score and (**B**) **a**crotime and digit symbol score. Each individual data point represents one participant. Lines represent the estimated change in the outcome for every unit change of the predictor. Shaded regions represent the 95% CI for these estimates. Beta coefficient (*b*) and unadjusted *P*-value (*P*) obtained from linear regression analysis adjusted for age, sex, *APOE4* carrier status, and years of education. Covariates were regressed out in the added variable plot. Acrotime is not translatable to military time due to regressing out the covariates. For reference, the mean and SD of acrotime are 14.26 (2:16 P.M.) and 1.53 (1 h and 32 min).

There was a positive association between acrotime and the CVLT short delay free recall total score [*b* = 0.73, 95% CI: (0.0059, 1.45), *P* = 0.048, FDR-adjusted *P* = 0.096, *n* = 67, Fig. 4A]. Additionally, there was a negative association between acrotime and the WAIS Digit Symbol Substitution score [*b* = −3.57, 95% CI: (−6.51, −0.64), *P* = 0.018, FDR-adjusted *P* = 0.072, *n* = 67, Fig. 4B]. These results indicate that an earlier peak activity time was associated with worse performance in verbal memory, but a later peak activity time was associated with worse performance in the Digit Symbol Substitution, a measure of attention/processing speed. IV was not associated with the CVLT short delay free recall total score or the WAIS Digit Symbol Substitution score.

### Tau mediates the association between acrotime and short delay verbal memory

**Figure 5:**
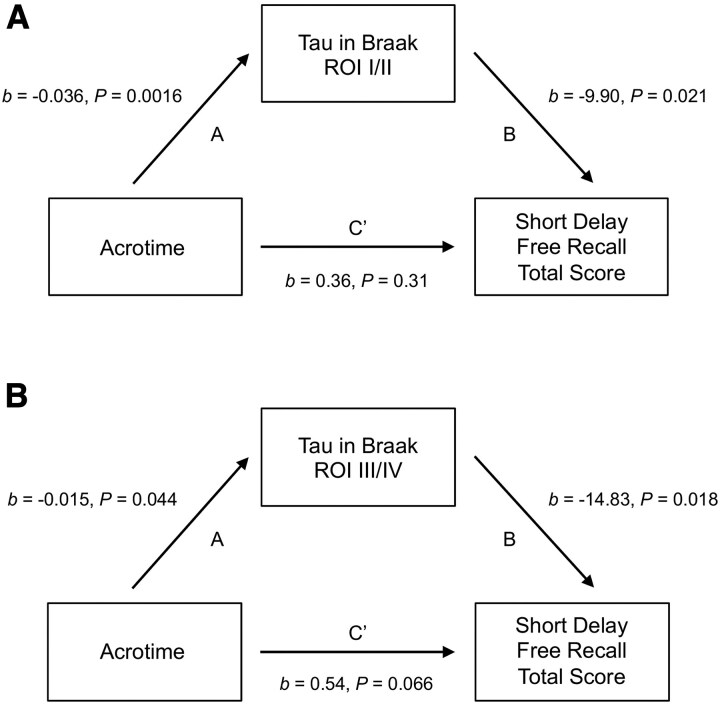
**Tau mediates the association between acrotime and short delay verbal memory.** Mediation model diagrams for the associations between acrotime and short delay free recall total score mediated by (**A**) tau in Braak ROIs I/II and (**B**) tau in Braak ROIs III/IV. Beta coefficients (*b*) and *P*-values (*P*) obtained from mediation analysis. All models were adjusted for age, sex, *APOE4* status, and years of education for a sample size of *n* = 66.

Exploratory mediation analysis (Table 2) identified two mediators in the relationship between acrotime and short delay verbal memory (Fig. 4A). We found statistically significant indirect effects of acrotime on short delay verbal memory via tau in Braak ROIs I/II [Total Indirect Effect (TIE) = 0.36, 95% CI: (0.014, 0.86), *P* = 0.040, *n* = 66, Fig. 5A] and III/IV [TIE = 0.23, 95% CI: (0.0064, 0.55), *P* = 0.044, *n* = 66, Fig. 5B]. These results indicate that the relationship between acrotime and short delay verbal memory was mediated by tau burden in ROIs I–IV. When the independent variable (acrotime) was swapped with the mediator (tau), acrotime did not exhibit any indirect mediating effects in the associations between tau in Braak ROIs I/II and short delay verbal memory, nor tau in Braak ROIs III/IV and short delay verbal memory.

**Table 2:** Mediation analysis results

Mediations	Estimate	95% CI	*P*
**Mediator: Tau in Braak ROIs I/II**			
Total effect	0.72	(0.077, 1.37)	0.029
Total direct effect	0.36	(−0.34, 1.09)	0.31
TIE	0.36	(0.014, 0.86)	0.040
Proportion mediated	0.50	(−0.044, 2.08)	0.064
**Mediator: Tau in Braak ROIs III/IV**			
Total effect	0.76	(0.11, 1.42)	0.018
Total direct effect	0.54	(−0.032, 1.16)	0.066
TIE	0.23	(0.0064, 0.55)	0.044
Proportion mediated	0.30	(−0.012, 0.99)	0.054

Mediation analysis results for the associations between acrotime and short delay free recall total score mediated by tau in Braak ROIs I/II and III/IV. All models were adjusted for age, sex, *APOE4* carrier status, and years of education for a sample size of *n* = 66.

Tau in Braak ROIs I/II and III/IV was not a mediator in the relationship between acrotime and attention/processing speed. Composite Aβ was not a mediator in the relationship between acrotime and short delay verbal memory, nor between acrotime and attention/processing speed.

## Discussion

This study reports associations between circadian rhythms, Alzheimer’s disease pathological biomarkers, and the cognitive domains of verbal memory and attention/processing speed in an older adult population with objective early cognitive impairment. Earlier circadian rhythm timing was associated with higher levels of Aβ, and this relationship was stronger in *APOE4* carriers compared with non-carriers. Earlier circadian rhythm timing and higher rhythm fragmentation were associated with higher levels of tau. The relationship between circadian rhythm fragmentation and tau strengthened with age and disproportionately affected men more than women. Earlier circadian rhythm timing was associated with worse verbal memory, whereas later circadian rhythm timing was associated with worse attention and processing speed. The relationship between circadian rhythm timing and verbal memory was mediated by tau levels.

Few studies have investigated circadian rhythms with respect to Aβ and tau neuropathology in early Alzheimer’s states, such as preclinical and MCI populations.^25,68-70^ To our knowledge, this is the first study to investigate the associations between circadian rhythms and pathological hallmarks (Aβ and tau) as well as cognition in older adults with objective early cognitive impairment. Moreover, it is the first to identify an Alzheimer’s pathological protein, tau, as a mediator in the relationship between circadian rhythms and cognition.

### Circadian rhythms and factors of age, sex, and *APOE4* carrier status

The correlation observed between older age and earlier circadian timing is in agreement with existing literature, as circadian phase advances are commonly observed with increasing age.^71^ The mechanisms underlying this shift are not fully understood, although one genome-wide transcriptomic study identified 1000 genes in the prefrontal cortex that exhibit age-dependent alterations in circadian rhythmicity and phase.^72^ Although our study did not observe a relationship between age and IV, two studies have reported this link in older adults that included participants aged 45 years and over.^25,73^ This discrepancy may be due to our more restricted age range of 55–80 years and smaller study sample size. Alternatively, our finding of a correlation between age and acrotime, but not age and IV, may indicate that changes to circadian timing occur earlier in the ageing process, prior to the emergence of changes in circadian fragmentation.

Similar to our findings, a recent 2021 study did not observe sex differences in circadian timing, but interaction analysis revealed that the association between an earlier circadian phase and age was stronger in men, indicating that men may be more prone to age-associated advanced circadian rhythms.^74^ Their study did, however, identify sex differences in circadian robustness, with men exhibiting higher IV than women. In agreement with our findings, another study did not observe differences between *APOE4* carriers and non-carriers in any circadian rhythm measures, including acrophase (analogous to acrotime) and IV.^25^

### Circadian rhythms and Aβ

Our finding that earlier circadian timing was associated with higher Aβ pathology is aligned with a separate study, which found that Aβ+ participants had higher levels of activity in the early morning through late afternoon compared to Aβ− individuals.^69^ Interestingly, these findings of an association between an advanced circadian phase and higher levels of Alzheimer’s pathology contrast with what is seen in patients with Alzheimer’s disease and dementia. Patients with Alzheimer’s disease typically present with delayed circadian phases, where activity is shifted towards the evening.^13,17-19^ However, studies in preclinical and MCI populations report mixed outcomes relating to both earlier and later circadian timing in association with MCI and/or risk of developing MCI and dementia.^23,28,30,75,76^ These variable findings signify that changes in circadian phase in either direction may increase the risk of developing MCI and Alzheimer’s disease.

Our observation that IV and composite Aβ were not associated is aligned with a separate study that reported no differences between Aβ+ and Aβ− preclinical older adults using standard sleep parameters, such as IV and acrophase.^69^ However, they did identify circadian variability differences between Aβ+ and Aβ− individuals that depended on the timing of the day when they used a unique measure of circadian variability calculated with a non-parametric technique called function-on-scalar regression. Another study similarly reported that preclinical older adult participants who were Aβ+ had significantly higher IV than Aβ− participants.^25^ The discrepancies between our results and these two studies may be due to differences in Aβ quantification, as we did not classify our participants into Aβ+ and Aβ− groups, but rather assessed Aβ SUVR continuously.

#### Mechanisms

There is strong mechanistic evidence that links circadian rhythms and Aβ in animal studies. A mouse model identified a circadian diurnal variation of interstitial fluid Aβ, where levels were highest during the wake phase and lowest during the sleep phase.^77^ Chronic sleep restriction of the mice accelerated Aβ plaque burden, whereas enhancing sleep by blocking orexin signalling (a regulator of wakefulness) inhibited Aβ accumulation. A different mouse study demonstrated that the targeted deletion of the circadian core clock gene *Bmal1* disrupted Aβ circadian oscillations in hippocampal interstitial fluid, and in turn accelerated the accumulation of Aβ plaques.^78^ These findings may be due to the clearance of metabolites and waste, including Aβ, that occurs in the brain during sleep.^79^

Similar findings exist in humans, who have exhibited diurnal oscillations of CSF Aβ, in which Aβ linearly increases throughout a day. These dynamics, however, were attenuated in those with greater Aβ deposition,^80^ which further supports an association between circadian disruption and Aβ pathology. Although evidence points to circadian dysfunction directly leading to Aβ dysregulation,^77-79,81^ it is possible that the resulting accumulation of Aβ would contribute to neuronal damage, which would in turn damage brain areas involved in the circadian system and further disrupt natural rhythms in a feed-forward loop.^82^

The specific mechanism supporting our findings may involve circadian regulation of immune cells that are responsible for Aβ waste clearance. A recent study reported that Aβ42 phagocytosis by macrophages in mice occurred on a daily rhythmic oscillation.^81^ This oscillation was due to the circadian regulation of cell surface molecules called heparan sulphate proteoglycans, which are molecules important to Aβ42 clearance. Ablating the heparan sulphate proteoglycans from macrophages caused the circadian oscillation of Aβ42 phagocytosis to disappear. This finding implicates the immune response as a central mechanism in the relationship between circadian disruption and Aβ accumulation in Alzheimer’s disease. Other proposed mechanisms that may explain circadian control of Aβ include circadian oscillations in neural activity, changes in slow wave sleep, and glymphatic system waste clearance of Aβ.^82^ These mechanisms may underlie the association we observed between earlier circadian timing and higher Aβ levels.

#### Interaction with *APOE4* carrier status

Little information is available on how circadian rhythms are involved with the *APOE* gene, but evidence exists that links *APOE4* carriers to sleep disruptions compared with non-carriers.^50,83,84^ Moreover, a mouse study found that deletion of the core clock gene *Bmal1* resulted in increased expression of the *APOE* gene.^78^ As *APOE4* has been shown to promote fibrillar Aβ plaque deposition,^85-87^ and Aβ deposition is more abundant in *APOE4* carriers than in non-carriers,^88^ increased *APOE* expression caused by circadian dysregulation could exacerbate Aβ pathology and explain the interaction observed in our study.

#### Circadian rhythms and tau

The relationship we observed between lower acrotime and higher tau was strongest and remained significant after FDR correction only in the earliest Braak ROIs I/II, where tau pathology begins in the transentorhinal cortex. In contrast to these findings, a separate similar study did not observe a relationship between acrophase and tau,^25^ possibly due to tau quantification differences, as they utilized the ratio of CSF phosphorylated tau_181_ to Aβ42 as a marker of Alzheimer’s-related neurodegeneration. The association between IV and tau in later Braak ROIs III/IV (limbic) remained significant after FDR correction, indicating that higher circadian fragmentation was significantly associated with higher tau pathological burden. This result aligns with a separate study that also identified a relationship between higher IV and a higher ratio of CSF phosphorylated tau_181_ to Aβ42.^25^

An advantage of our study is the use of PET imaging to quantify tau. As neurofibrillary tangles directly correlate with the presence and severity of dementia in Alzheimer’s disease,^89^ tau PET in adults with early cognitive impairment allows the assessment of early pathological changes that may be predictive of future cognitive decline into MCI or Alzheimer’s disease. Indeed, tau PET has been shown to predict cognitive decline in individuals who are cognitively normal^90,91^ and follows a topographic, Braak staging that informs on pathological tau spread across distinct anatomical brain areas, reflecting clinical manifestations. The successive stages of Braak staging are described elsewhere,^92^ but the stages that were analysed in the present study can be summarized as transentorhinal (I/II) and limbic (III/IV). Our findings reveal that acrotime was most strongly associated with tau in the earliest Braak ROIs I/II, whereas IV was associated with tau only in later Braak ROIs III/IV. Together, these results may suggest that circadian phase advances contribute to tau accumulation in brain areas that are affected in the earliest stages of typical tau progression in Alzheimer’s disease, whereas circadian fragmentation contributes to tau accumulation in brain areas that are affected in later stages. As such, circadian phase shifting earlier may be the earliest circadian indicator of tau pathological spreading, and circadian fragmentation may appear later in the disease trajectory. Older adults with an earlier circadian phase may be at risk for tau spread and cognitive decline, and may benefit from circadian therapies to regulate circadian phase.

#### Mechanisms

Tau phosphorylation in mice has been demonstrated to follow a circadian rhythm that depends on body temperature, with tau being hyperphosphorylated during sleep.^93^ In a transgenic mouse model of Alzheimer’s disease, irregular sleep–wake cycles induced by sleep deprivation resulted in dysfunctional tau metabolism.^94^ One study identified a mechanistic link between altered circadian function and tau aggravation. Using a tau transgenic mouse model, they found that circadian regulation by the core clock gene *Bmal1* modulated the oscillation of a chaperone protein called Hsp70, which plays a crucial role in tau metabolism.^95^ A different study using a mouse model of Alzheimer’s disease demonstrated that melatonin administration reduced expression levels of phosphorylated tau,^96^ introducing circadian therapy as a viable possibility for the attenuation of Alzheimer’s-related tau neuropathology. Evidence supports a bi-directional relationship in which tau accumulation can also damage circadian rhythms. A study found that the Tg4510 mouse model of tauopathy exhibited a longer circadian period, tauopathy in the suprachiasmatic nucleus, and disrupted circadian cyclical expression of core clock genes in the hypothalamus and hippocampus.^97^ Together, these mouse studies help explain the relationship we observed between circadian timing/fragmentation and tau pathological burden.

#### Interactions with age and sex

IV has been shown to be associated with older age,^25,45,73^ and ageing is the most prominent risk factor for Alzheimer’s disease, which together may explain the age modification effect we observed in the relationships between IV and tau.

The relationships between IV and tau were largely driven by men. In another study in older adults, participants with low circadian amplitude (lower circadian strength) had an increased risk of developing Alzheimer’s, and this association was stronger in men than in women,^76^ similar to our sex-specific findings. A mechanistic explanation for these sex differences could be discrepancies in hormone production between men and women. Numerous studies have identified reductions in melatonin in Alzheimer’s disease,^4-10^ and melatonin has been shown to efficiently attenuate tau hyperphosphorylation.^98^ As women exhibit significantly higher melatonin amplitudes than men,^99^ lower melatonin levels in men may impact circadian rhythms and exacerbate tau neuropathology.

### Circadian rhythms and cognition

Across the literature, variable findings between circadian timing and cognition are observed in older adults. Using various actigraphy measures of circadian timing and cognitive assessments, studies report that both circadian rhythm phase advances^24,75^ and delays^26,70,100^ are associated with worse performance across numerous cognitive domains. Evidently, changes in circadian phase in either direction away from normal are related to different aspects of poorer cognitive performance, similar to how both phase advances and delays are seen in MCI and Alzheimer’s disease. Circadian advances and delays may differentially impact Alzheimer’s pathological processes and, in turn, affect separate cognitive domains.

Although our study did not observe a relationship between IV and cognition, other studies have found that a number of circadian measures quantifying lower rhythm robustness were associated with worse cognition in preclinical and MCI older adult populations.^24,27-29,101^ As our study’s participants were experiencing very early cognitive changes, our observation of a link between circadian timing and cognition, but not between circadian fragmentation and cognition, may further support the idea that a change in timing is the earliest circadian indicator of Alzheimer’s disease pathological progression and a predictor of future cognitive decline.

#### Mediations

The relationship between acrotime and short delay verbal memory was mediated by tau pathological burden in Braak ROIs I–IV. This study is, to our knowledge, the first to identify a mechanistic mediation between circadian rhythms, a pathological hallmark of Alzheimer’s disease (tau), and cognition. A related study identified a metabolic link, showing that cerebral glucose metabolism mediated the relationship between acrotime and global cognition using the Mini-Mental State Examination.^70^

#### Mechanisms

The relationships observed between acrotime and cognition are likely mediated by underlying molecular and cellular pathological processes that contribute to neuronal degeneration in the brain, which eventually leads to cognitive impairment. Our work identified tau accumulation in the transentorhinal and limbic cortices as two such mediators. Several papers provide mechanistic explanations for circadian regulation of tau metabolism and aggregation,^93-96^ which could in turn exacerbate neurodegeneration and eventually lead to cognitive decline. As circadian rhythms exert oscillatory control over a significant number of processes in all organs and tissues throughout the body, there are likely numerous other mediators through which circadian dysfunction leads to reduced cognition. Hypothesized mechanisms include Aβ aggregation and metabolism, cholinergic disturbances, loss of retinal ganglion cells, melatonin loss, neuronal homeostasis, oxidative stress, inflammatory processes, vascular dysfunction, metabolic dysfunction, and glymphatic clearance.^102-105^

### Limitations

The relatively modest sample size of this study produced marginally significant interaction findings and associations between circadian rhythms and cognition. As such, these findings are exploratory and hypothesis-generating, providing information on the directionality of trends that should guide future research in larger older adult cohorts. The majority of our study sample consisted of self-reported White, highly educated individuals, which limits generalizability to other populations. The small number of *APOE4* carriers (*n* = 18) further limits the interpretation of findings involving *APOE4* carrier status. Moreover, our study used cross-sectional data, limiting interpretations of longitudinal change and causality. Although this work focuses on circadian rhythms as the predictor, we cannot exclude the possibility that Aβ and tau pathological processes damage brain areas involved in circadian regulation, which would also explain our findings. However, as our mediation analyses did not identify indirect effects when the independent variables and mediators were swapped, our findings suggest that circadian rhythms are at the forefront of these relationships and Alzheimer’s disease-related pathological progression. This interpretation is supported by substantial evidence in animal models that circadian rhythms regulate Aβ^77,78,81^ and tau,^93-96^ further supporting the view that circadian dysfunction is an early indicator and potential contributor to neuropathology, rather than a consequence.

### Future directions

In order to determine the cause-and-effect relationship between circadian rhythms and Alzheimer’s biomarkers, future longitudinal studies should confirm our findings in a larger cohort of preclinical older adults prior to the onset of neuropathology. This work would evaluate whether circadian dysfunction precedes Aβ and tau buildup, as well as cognitive decline. Moreover, studies should assess other possible mediators of the relationships between circadian rhythms and cognition. Randomized controlled trials should utilize circadian-based interventions (timed melatonin administration, bright light therapy, physical therapy, proper sleep hygiene, regulation of feeding, and exercise schedule) in patients with objective early cognitive impairment or other Alzheimer’s risk factors to assess the potential of circadian therapy in attenuating Alzheimer’s-related outcomes.

## Conclusion

This study revealed that circadian rhythm timing and fragmentation were associated with Aβ, tau, and cognition in older adults with early cognitive impairment. As one of the only studies investigating these relationships in this population, we provide evidence for tau as a biological mediator in the relationship between circadian timing and cognition. Older adults with variable circadian phases and more fragmented rhythms may be at higher risk for developing Alzheimer’s pathological hallmarks and cognitive impairment. This risk appears to increase with age and disproportionately affect men and *APOE4* carriers. This study identified circadian rhythms as potential modifiable risk factors that could be targeted with circadian-based therapies to attenuate pathological progression of Aβ and tau in the brain, and in turn prevent or slow the transition into MCI and dementia for at-risk older adults.

## Supplementary Material

fcaf322_Supplementary_Data

## Data Availability

All LEARNit data reported in this study will be shared by the lead contact, Judy Pa (jpa@ucsd.edu), upon a data request through a data use agreement. All original code has been deposited at Harvard Dataverse at https://doi.org/10.7910/DVN/2KKUS1 and is publicly available as of the date of the publication. Any additional information required to reanalyse the data reported in this paper is available from the lead contact upon request.

## References

[1] Zeitzer JM, Dijk DJ, Kronauer RE, Brown EN, Czeisler CA. Sensitivity of the human circadian pacemaker to nocturnal light: Melatonin phase resetting and suppression. J Physiol. 2000;526(Pt 3):695. 10922269 10.1111/j.1469-7793.2000.00695.x PMC2270041

[2] Cajochen C, Zeitzer JM, Czeisler CA, Dijk DJ. Dose-response relationship for light intensity and ocular and electroencephalographic correlates of human alertness. Behav Brain Res. 2000;115(1):75–83. 10996410 10.1016/s0166-4328(00)00236-9

[3] O’Byrne NA, Yuen F, Butt WZ, Liu PY. Sleep and circadian regulation of cortisol: A short review. Curr Opin Endocr Metab Res. 2021;18:178–186. 35128146 10.1016/j.coemr.2021.03.011 PMC8813037

[4] Skene DJ, Vivien-Roels B, Sparks DL, et al Daily variation in the concentration of melatonin and 5-methoxytryptophol in the human pineal gland: Effect of age and Alzheimer’s disease. Brain Res. 1990;528(1):170–174. 2245336 10.1016/0006-8993(90)90214-v

[5] Uchida K, Okamoto N, Ohara K, Morita Y. Daily rhythm of serum melatonin in patients with dementia of the degenerate type. Brain Res. 1996;717(1):154–159. 8738265 10.1016/0006-8993(96)00086-8

[6] Liu RY, Zhou JN, van Heerikhuize J, Hofman MA, Swaab DF. Decreased melatonin levels in postmortem cerebrospinal fluid in relation to aging, Alzheimer’s disease, and apolipoprotein E-ε4/4 genotype. J Clin Endocrinol Metab. 1999;84(1):323–327. 9920102 10.1210/jcem.84.1.5394

[7] Mishima K, Tozawa T, Satoh K, Matsumoto Y, Hishikawa Y, Okawa M. Melatonin secretion rhythm disorders in patients with senile dementia of Alzheimer’s type with disturbed sleep–waking. Biol Psychiatry. 1999;45(4):417–421. 10071710 10.1016/s0006-3223(97)00510-6

[8] Ohashi Y, Okamoto N, Uchida K, Iyo M, Mori N, Morita Y. Daily rhythm of serum melatonin levels and effect of light exposure in patients with dementia of the Alzheimer’s type. Biol Psychiatry. 1999;45(12):1646–1652. 10376127 10.1016/s0006-3223(98)00255-8

[9] Zhou JN, Liu RY, Kamphorst W, Hofman MA, Swaab DF. Early neuropathological Alzheimer’s changes in aged individuals are accompanied by decreased cerebrospinal fluid melatonin levels. J Pineal Res. 2003;35(2):125–130. 12887656 10.1034/j.1600-079x.2003.00065.x

[10] Wu YH, Zhou JN, Van Heerikhuize J, Jockers R, Swaab DF. Decreased MT1 melatonin receptor expression in the suprachiasmatic nucleus in aging and Alzheimer’s disease. Neurobiol Aging. 2007;28(8):1239–1247. 16837102 10.1016/j.neurobiolaging.2006.06.002

[11] Ouanes S, Popp J. High cortisol and the risk of dementia and Alzheimer’s disease: A review of the literature. Front Aging Neurosci. 2019;11:43. 30881301 10.3389/fnagi.2019.00043 PMC6405479

[12] Witting W, Kwa IH, Eikelenboom P, Mirmiran M, Swaab DF. Alterations in the circadian rest-activity rhythm in aging and Alzheimer’s disease. Biol Psychiatry. 1990;27(6):563–572. 2322616 10.1016/0006-3223(90)90523-5

[13] Satlin A, Volicer L, Stopa EG, Harper D. Circadian locomotor activity and core-body temperature rhythms in Alzheimer’s disease. Neurobiol Aging. 1995;16(5):765–771. 8532109 10.1016/0197-4580(95)00059-n

[14] Hatfield CF, Herbert J, van Someren EJW, Hodges JR, Hastings MH. Disrupted daily activity/rest cycles in relation to daily cortisol rhythms of home-dwelling patients with early Alzheimer’s dementia. Brain. 2004;127(5):1061–1074. 14998915 10.1093/brain/awh129

[15] Hooghiemstra AM, Eggermont LHP, Scheltens P, van der Flier WM, Scherder EJA. The rest-activity rhythm and physical activity in early-onset dementia. Alzheimer Dis Assoc Disord. 2015;29(1):45. 24632989 10.1097/WAD.0000000000000037

[16] van Someren EJW, Hagebeuk EEO, Lijzenga C, et al Circadian rest—Activity rhythm disturbances in Alzheimer’s disease. Biol Psychiatry. 1996;40(4):259–270. 8871772 10.1016/0006-3223(95)00370-3

[17] Volicer L, Harper DG, Manning BC, Goldstein R, Satlin A. Sundowning and circadian rhythms in Alzheimer’s disease. Am J Psychiatry. 2001;158(5):704–711. 11329390 10.1176/appi.ajp.158.5.704

[18] Ancoli-Israel S, Klauber MR, Jones DW, et al Variations in circadian rhythms of activity, sleep, and light exposure related to dementia in nursing-home patients. Sleep. 1997;20(1):18–23. 9130329

[19] Harper DG, Stopa EG, McKee AC, et al Differential circadian rhythm disturbances in men with Alzheimer disease and frontotemporal degeneration. Arch Gen Psychiatry. 2001;58(4):353–360. 11296096 10.1001/archpsyc.58.4.353

[20] Okawa M, Mishima K, Hishikawa Y, Hozumi S, Hori H, Takahashi K. Circadian rhythm disorders in sleep-waking and body temperature in elderly patients with dementia and their treatment. Sleep. 1991;14(6):478–485. 1798879 10.1093/sleep/14.6.478

[21] Harper DG, Volicer L, Stopa EG, McKee AC, Nitta M, Satlin A. Disturbance of endogenous circadian rhythm in aging and Alzheimer disease. Am J Geriatr Psychiatry. 2005;13(5):359–368. 15879584 10.1176/appi.ajgp.13.5.359

[22] Bliwise DL, Carroll JS, Lee KA, Nekich JC, Dement WC. Sleep and “sundowning” in nursing home patients with dementia. Psychiatry Res. 1993;48(3):277–292. 8272449 10.1016/0165-1781(93)90078-u

[23] Ortiz-Tudela E, Martinez-Nicolas A, Díaz-Mardomingo C, et al The characterization of biological rhythms in mild cognitive impairment. BioMed Res Int. 2014;2014(1):524971. 25157363 10.1155/2014/524971 PMC4124835

[24] Rogers-Soeder TS, Blackwell T, Yaffe K, et al Rest-activity rhythms and cognitive decline in older men: The osteoporotic fractures in men sleep study. J Am Geriatr Soc. 2018;66(11):2136–2143. 30136716 10.1111/jgs.15555 PMC6235690

[25] Musiek ES, Bhimasani M, Zangrilli MA, Morris JC, Holtzman DM, Ju YES. Circadian rest-activity pattern changes in aging and preclinical Alzheimer disease. JAMA Neurol. 2018;75(5):582–590. 29379963 10.1001/jamaneurol.2017.4719 PMC5885197

[26] Yi Lee PM, Ling Kwok BH, Ting Ma JY, Tse LA. A population-based prospective study on rest-activity rhythm and mild cognitive impairment among Hong Kong healthy community-dwelling older adults. Neurobiol Sleep Circadian Rhythms. 2021;10:100065. 33997474 10.1016/j.nbscr.2021.100065 PMC8091051

[27] Alfini A, Albert M, Faria AV, et al Associations of actigraphic sleep and circadian rest/activity rhythms with cognition in the early phase of Alzheimer’s disease. SLEEP Adv. 2021;2(1):zpab007. 10.1093/sleepadvances/zpab007 PMC8168567 34095836

[28] Xiao Q, Shadyab AH, Rapp SR, et al Rest-activity rhythms and cognitive impairment and dementia in older women: Results from the women’s health initiative. J Am Geriatr Soc. 2022;70(10):2925–2937. 35708069 10.1111/jgs.17926 PMC9588636

[29] Kim SJ, Lee JH, Jang JW, Jung HS, Suh IB. Abnormalities of rest-activity and light exposure rhythms associated with cognitive function in patients with mild cognitive impairment (MCI). J Circadian Rhythms. 2023;21(1):4. 38162255 10.5334/jcr.227 PMC10756154

[30] Tranah GJ, Blackwell T, Stone KL, et al Circadian activity rhythms and risk of incident dementia and mild cognitive impairment in older women. Ann Neurol. 2011;70(5):722–732. 22162057 10.1002/ana.22468 PMC3244839

[31] The R Foundation . The R Project for Statistical Computing. https://www.r-project.org/. Accessed 23 October 2024.

[32] ActivInsights . GENEActiv: Raw Data Accelerometer. https://activinsights.com/technology/geneactiv/. Accessed 23 October 2024.

[33] van Hees VT, Migueles JH, Sabia S, et al GGIR: Raw Accelerometer Data Analysis. https://cran.r-project.org/web/packages/GGIR/index.html. Accessed 23 October 2024.

[34] Migueles JH, Rowlands AV, Huber F, Sabia S, van Hees VT. GGIR: A research community–driven open source R package for generating physical activity and sleep outcomes from multi-day raw accelerometer data. J Meas Phys Behav. 2019;2(3):188–196.

[35] van Hees VT, Gorzelniak L, León ECD, et al Separating movement and gravity components in an acceleration signal and implications for the assessment of human daily physical activity. PLoS One. 2013;8(4):e61691. 23626718 10.1371/journal.pone.0061691 PMC3634007

[36] Monk TH, Aplin LC. Spring and autumn daylight saving time changes: Studies of adjustment in sleep timings, mood, and efficiency. Ergonomics. 1980;23(2):167–178. 7398616 10.1080/00140138008924730

[37] Ancoli-Israel S, Cole R, Alessi C, Chambers M, Moorcroft W, Pollak CP. The role of actigraphy in the study of sleep and circadian rhythms. Sleep. 2003;26(3):342–392. 12749557 10.1093/sleep/26.3.342

[38] Cornelissen G . Cosinor-based rhythmometry. Theor Biol Med Model. 2014;11(1):16. 24725531 10.1186/1742-4682-11-16 PMC3991883

[39] Martin J, Marler M, Shochat T, Ancoli-Israel S. Circadian rhythms of agitation in institutionalized patients with Alzheimer’s disease. Chronobiol Int. 2000;17(3):405–418. 10841213 10.1081/cbi-100101054

[40] McDonnell EI, Zipunnikov V, Schrack JA, Goldsmith J, Wrobel J. Registration of 24-hour accelerometric rest-activity profiles and its application to human chronotypes. Biol Rhythm Res. 2022;53(8):1299–1319. 35784395 10.1080/09291016.2021.1929673 PMC9246189

[41] Di J, Zipunnikov V, van Hees VT. ActCR: Extract Circadian Rhythms Metrics from Actigraphy Data. https://cran.r-project.org/web/packages/ActCR/index.html. Accessed 23 October 2024.

[42] Di J, Spira A, Bai J, et al Joint and individual representation of domains of physical activity, sleep, and circadian rhythmicity. Stat Biosci. 2019;11(2):371–402. 32440309 10.1007/s12561-019-09236-4 PMC7241438

[43] Schabus CBNSM. nparACT: Non-Parametric Measures of Actigraphy Data. https://cran.r-project.org/web/packages/nparACT/index.html. Accessed 23 October 2024.

[44] Blume C, Santhi N, Schabus M. nparACT’ package for R: A free software tool for the non-parametric analysis of actigraphy data. MethodsX. 2016;3:430–435. 27294030 10.1016/j.mex.2016.05.006 PMC4890079

[45] Huang YL, Liu RY, Wang QS, Van Someren EJW, Xu H, Zhou JN. Age-associated difference in circadian sleep–wake and rest–activity rhythms. Physiol Behav. 2002;76(4):597–603. 12126998 10.1016/s0031-9384(02)00733-3

[46] Someren EJWV, Kessler A, Mirmiran M, Swaab DF. Indirect bright light improves circadian rest-activity rhythm disturbances in demented patients. Biol Psychiatry. 1997;41(9):955–963. 9110101 10.1016/S0006-3223(97)89928-3

[47] Van Someren EJW, Swaab DF, Colenda CC, Cohen W, McCall WV, Rosenquist PB. Bright light therapy: Improved sensitivity to its effects on rest-activity rhythms in Alzheimer patients by application of nonparametric methods. Chronobiol Int. 1999;16(4):505–518. 10442243 10.3109/07420529908998724

[48] Scherder EJA, Van Someren EJW, Swaab DF. Transcutaneous electrical nerve stimulation (TENS) improves the rest–activity rhythm in midstage Alzheimer’s disease. Behav Brain Res. 1999;101(1):105–107. 10342404 10.1016/s0166-4328(98)00150-8

[49] Thoen E . padr: Quickly Get Datetime Data Ready for Analysis. https://cran.r-project.org/web/packages/padr/index.html. Accessed 23 October 2024.

[50] Fenton L, Isenberg AL, Aslanyan V, et al Variability in objective sleep is associated with Alzheimer’s pathology and cognition. Brain Commun. 2023;5(2):fcad031. 36895954 10.1093/braincomms/fcad031 PMC9989141

[51] Albrecht D, Isenberg AL, Stradford J, et al Associations between vascular function and tau PET are associated with global cognition and amyloid. J Neurosci. 2020;40(44):8573–8586. 33046556 10.1523/JNEUROSCI.1230-20.2020 PMC7605425

[52] Albrecht DS, Sagare A, Pachicano M, et al Early neuroinflammation is associated with lower amyloid and tau levels in cognitively normal older adults. Brain Behav Immun. 2021;94:299–307. 33486003 10.1016/j.bbi.2021.01.010 PMC8793040

[53] Avants BB, Yushkevich P, Pluta J, et al The optimal template effect in hippocampus studies of diseased populations. NeuroImage. 2010;49(3):2457–2466. 19818860 10.1016/j.neuroimage.2009.09.062 PMC2818274

[54] Avants BB, Tustison NJ, Song G, Cook PA, Klein A, Gee JC. A reproducible evaluation of ANTs similarity metric performance in brain image registration. NeuroImage. 2011;54(3):2033–2044. 20851191 10.1016/j.neuroimage.2010.09.025 PMC3065962

[55] Laboratories for Computational Neuroimaging . FreeSurfer. https://surfer.nmr.mgh.harvard.edu. Accessed 23 October 2024.

[56] Baker SL, Maass A, Jagust WJ. Considerations and code for partial volume correcting [18F]-AV-1451 tau PET data. Data Brief. 2017;15:648–657. 29124088 10.1016/j.dib.2017.10.024 PMC5671473

[57] Vemuri P, Lowe VJ, Knopman DS, et al Tau-PET uptake: Regional variation in average SUVR and impact of amyloid deposition. Alzheimers Dement Diagn Assess Dis Monit. 2017;6(1):21–30. 10.1016/j.dadm.2016.12.010 PMC5257031 28138510

[58] Diedrichsen J . A spatially unbiased atlas template of the human cerebellum. NeuroImage. 2006;33(1):127–138. 16904911 10.1016/j.neuroimage.2006.05.056

[59] Diedrichsen J, Balsters JH, Flavell J, Cussans E, Ramnani N. A probabilistic MR atlas of the human cerebellum. NeuroImage. 2009;46(1):39–46. 19457380 10.1016/j.neuroimage.2009.01.045

[60] Diedrichsen J, Maderwald S, Küper M, et al Imaging the deep cerebellar nuclei: A probabilistic atlas and normalization procedure. NeuroImage. 2011;54(3):1786–1794. 20965257 10.1016/j.neuroimage.2010.10.035

[61] Diedrichsen J, Zotow E. Surface-based display of volume-averaged cerebellar imaging data. PLoS One. 2015;10(7):e0133402. 26230510 10.1371/journal.pone.0133402 PMC4521932

[62] Landau S, Jagust W. Alzheimer’s Disease Neuroimaging Initiative. Florbetapir processing methods. https://adni.bitbucket.io/reference/docs/UCBERKELEYAV45/ADNI_AV45_Methods_JagustLab_06.25.15.pdf. Accessed 23 October 2024.

[63] Braak H, Braak E. Neuropathological stageing of Alzheimer-related changes. Acta Neuropathol. 1991;82(4):239–259. 1759558 10.1007/BF00308809

[64] Schöll M, Lockhart SN, Schonhaut DR, et al PET imaging of tau deposition in the aging human brain. Neuron. 2016;89(5):971–982. 26938442 10.1016/j.neuron.2016.01.028 PMC4779187

[65] Woods SP, Delis DC, Scott JC, Kramer JH, Holdnack JA. The California verbal learning test—Second edition: Test-retest reliability, practice effects, and reliable change indices for the standard and alternate forms. Arch Clin Neuropsychol. 2006;21(5):413–420. 16843636 10.1016/j.acn.2006.06.002

[66] Jaeger J . Digit symbol substitution test: The case for sensitivity over specificity in neuropsychological testing. J Clin Psychopharmacol. 2018;38(5):513. 30124583 10.1097/JCP.0000000000000941 PMC6291255

[67] Tingley D, Yamamoto T, Hirose K, et al mediation: Causal Mediation Analysis. https://cran.r-project.org/web/packages/mediation/index.html. Accessed 23 October 2024.

[68] Ju YES, McLeland JS, Toedebusch CD, et al Sleep quality and preclinical Alzheimer disease. JAMA Neurol. 2013;70(5):587–593. 23479184 10.1001/jamaneurol.2013.2334 PMC3676720

[69] Spira AP, Zipunnikov V, Raman R, et al Brain amyloid burden, sleep, and 24-hour rest/activity rhythms: Screening findings from the anti-amyloid treatment in asymptomatic Alzheimer’s and longitudinal evaluation of amyloid risk and neurodegeneration studies. SLEEP Adv. 2021;2(1):zpab015. 34661109 10.1093/sleepadvances/zpab015 PMC8519157

[70] Jeon SY, Byun MS, Yi D, et al Circadian rest-activity rhythm and longitudinal brain changes underlying late-life cognitive decline. Psychiatry Clin Neurosci. 2023;77(4):205–212. 36527292 10.1111/pcn.13521 PMC10360409

[71] Duffy JF, Zitting KM, Chinoy ED. Aging and circadian rhythms. Sleep Med Clin. 2015;10(4):423–434. 26568120 10.1016/j.jsmc.2015.08.002 PMC4648699

[72] Chen CY, Logan RW, Ma T, et al Effects of aging on circadian patterns of gene expression in the human prefrontal cortex. Proc Natl Acad Sci U S A. 2016;113(1):206–211. 26699485 10.1073/pnas.1508249112 PMC4711850

[73] Luik AI, Zuurbier LA, Hofman A, Van Someren EJW, Tiemeier H. Stability and fragmentation of the activity rhythm across the sleep-wake cycle: The importance of age, lifestyle, and mental health. Chronobiol Int. 2013;30(10):1223–1230. 23971909 10.3109/07420528.2013.813528

[74] Li J, Somers VK, Lopez-Jimenez F, Di J, Covassin N. Demographic characteristics associated with circadian rest-activity rhythm patterns: A cross-sectional study. Int J Behav Nutr Phys Act. 2021;18(1):107. 34407852 10.1186/s12966-021-01174-z PMC8371768

[75] Naismith SL, Hickie IB, Terpening Z, et al Circadian misalignment and sleep disruption in mild cognitive impairment. J Alzheimers Dis. 2014;38(4):857–866. 24100124 10.3233/JAD-131217

[76] Li P, Gao L, Gaba A, et al Circadian disturbances in Alzheimer’s disease progression: A prospective observational cohort study of community-based older adults. Lancet Healthy Longev. 2020;1(3):e96–e105. 34179863 10.1016/s2666-7568(20)30015-5 PMC8232345

[77] Kang JE, Lim MM, Bateman RJ, et al Amyloid-β dynamics are regulated by orexin and the sleep-wake cycle. Science. 2009;326(5955):1005–1007. 19779148 10.1126/science.1180962 PMC2789838

[78] Kress GJ, Liao F, Dimitry J, et al Regulation of amyloid-β dynamics and pathology by the circadian clock. J Exp Med. 2018;215(4):1059–1068. 29382695 10.1084/jem.20172347 PMC5881473

[79] Xie L, Kang H, Xu Q, et al Sleep drives metabolite clearance from the adult brain. Science. 2013;342(6156):373–377. 24136970 10.1126/science.1241224 PMC3880190

[80] Huang Y, Potter R, Sigurdson W, et al Effects of age and amyloid deposition on Aβ dynamics in the human central nervous system. Arch Neurol. 2012;69(1):51–58. 21911660 10.1001/archneurol.2011.235 PMC3254706

[81] Clark GT, Yu Y, Urban CA, et al Circadian control of heparan sulfate levels times phagocytosis of amyloid beta aggregates. PLOS Genet. 2022;18(2):e1009994. 35143487 10.1371/journal.pgen.1009994 PMC8830681

[82] Musiek ES, Xiong DD, Holtzman DM. Sleep, circadian rhythms, and the pathogenesis of Alzheimer disease. Exp Mol Med. 2015;47(3):e148–e148. 25766617 10.1038/emm.2014.121 PMC4351409

[83] Yesavage JA, Friedman L, Kraemer H, et al Sleep/wake disruption in Alzheimer’s disease: APOE status and longitudinal course. J Geriatr Psychiatry Neurol. 2004;17(1):20–24. 15018693 10.1177/0891988703261994

[84] Blackman J, Love S, Sinclair L, Cain R, Coulthard E. APOE ε4, Alzheimer’s disease neuropathology and sleep disturbance, in individuals with and without dementia. Alzheimers Res Ther. 2022;14(1):47. 35354468 10.1186/s13195-022-00992-y PMC8969347

[85] Holtzman DM, Fagan AM, Mackey B, et al Apolipoprotein E facilitates neuritic and cerebrovascular plaque formation in an Alzheimer’s disease model. Ann Neurol. 2000;47(6):739–747. 10852539

[86] Fagan AM, Watson M, Parsadanian M, Bales KR, Paul SM, Holtzman DM. Human and murine ApoE markedly alters Aβ metabolism before and after plaque formation in a mouse model of Alzheimer’s disease. Neurobiol Dis. 2002;9(3):305–318. 11950276 10.1006/nbdi.2002.0483

[87] Kim J, Jiang H, Park S, et al Haploinsufficiency of human APOE reduces amyloid deposition in a mouse model of amyloid-β amyloidosis. J Neurosci. 2011;31(49):18007–18012. 22159114 10.1523/JNEUROSCI.3773-11.2011 PMC3257514

[88] Kok E, Haikonen S, Luoto T, et al Apolipoprotein E–dependent accumulation of Alzheimer disease–related lesions begins in middle age. Ann Neurol. 2009;65(6):650–657. 19557866 10.1002/ana.21696

[89] Arriagada PV, Growdon JH, Hedley-Whyte ET, Hyman BT. Neurofibrillary tangles but not senile plaques parallel duration and severity of Alzheimer’s disease. Neurology. 1992;42(3):631–631. 1549228 10.1212/wnl.42.3.631

[90] Ossenkoppele R, Pichet Binette A, Groot C, et al Amyloid and tau PET-positive cognitively unimpaired individuals are at high risk for future cognitive decline. Nat Med. 2022;28(11):2381–2387. 36357681 10.1038/s41591-022-02049-x PMC9671808

[91] Strikwerda-Brown C, Hobbs DA, Gonneaud J, et al Association of elevated amyloid and tau positron emission tomography signal with near-term development of Alzheimer disease symptoms in older adults without cognitive impairment. JAMA Neurol. 2022;79(10):975–985. 35907254 10.1001/jamaneurol.2022.2379 PMC9339146

[92] Macedo AC, Tissot C, Therriault J, et al The use of tau PET to stage Alzheimer disease according to the Braak staging framework. J Nucl Med. 2023;64(8):1171–1178. 37321820 10.2967/jnumed.122.265200 PMC10394315

[93] Guisle I, Gratuze M, Petry S, et al Circadian and sleep/wake-dependent variations in tau phosphorylation are driven by temperature. Sleep. 2020;43(4):zsz266. 31702011 10.1093/sleep/zsz266

[94] Di Meco A, Joshi YB, Praticò D. Sleep deprivation impairs memory, tau metabolism, and synaptic integrity of a mouse model of Alzheimer’s disease with plaques and tangles. Neurobiol Aging. 2014;35(8):1813–1820. 24629673 10.1016/j.neurobiolaging.2014.02.011

[95] Han SM, Jang YJ, Kim EY, Park SA. The change in circadian rhythms in P301S transgenic mice is linked to variability in Hsp70-related tau disaggregation. Exp Neurobiol. 2022;31(3):196–207. 35786641 10.5607/en22019 PMC9272121

[96] Gong YH, Hua N, Zang X, Huang T, He L. Melatonin ameliorates Aβ1-42-induced Alzheimer’s cognitive deficits in mouse model. J Pharm Pharmacol. 2018;70(1):70–80. 28994117 10.1111/jphp.12830

[97] Stevanovic K, Yunus A, Joly-Amado A, et al Disruption of normal circadian clock function in a mouse model of tauopathy. Exp Neurol. 2017;294:58–67. 28461004 10.1016/j.expneurol.2017.04.015

[98] Lin L, Huang QX, Yang SS, Chu J, Wang JZ, Tian Q. Melatonin in Alzheimer’s disease. Int J Mol Sci. 2013;14(7):14575–14593. 23857055 10.3390/ijms140714575 PMC3742260

[99] Cain SW, Dennison CF, Zeitzer JM, et al Sex differences in phase angle of entrainment and melatonin amplitude in humans. J Biol Rhythms. 2010;25(4):288–296. 20679498 10.1177/0748730410374943 PMC3792014

[100] Walsh CM, Blackwell T, Tranah GJ, et al Weaker circadian activity rhythms are associated with poorer executive function in older women. Sleep. 2014;37(12):2009–2016. 25337947 10.5665/sleep.4260 PMC4548515

[101] Luik AI, Zuurbier LA, Hofman A, Van Someren EJW, Ikram MA, Tiemeier H. Associations of the 24-h activity rhythm and sleep with cognition: A population-based study of middle-aged and elderly persons. Sleep Med. 2015;16(7):850–855. 26028055 10.1016/j.sleep.2015.03.012

[102] Musiek ES, Holtzman DM. Mechanisms linking circadian clocks, sleep, and neurodegeneration. Science. 2016;354(6315):1004–1008. 27885006 10.1126/science.aah4968 PMC5219881

[103] Musiek ES . Circadian rhythms in AD pathogenesis: A critical appraisal. Curr Sleep Med Rep. 2017;3(2):85–92. 29308355 10.1007/s40675-017-0072-5 PMC5754029

[104] Homolak J, Mudrovčić M, Vukić B, Toljan K. Circadian rhythm and Alzheimer’s disease. Med Sci. 2018;6(3):52. 10.3390/medsci6030052 PMC6164904 29933646

[105] Uddin M, Tewari D, Mamun AA, et al Circadian and sleep dysfunction in Alzheimer’s disease. Ageing Res Rev. 2020;60:101046. 32171783 10.1016/j.arr.2020.101046

