# Supplementary material for: Circadian rhythms are associated with higher amyloid-β and tau and poorer cognition in older adults
Source: Brain Commun. 2025 Sep 8;7(5):fcaf322. doi: 10.1093/braincomms/fcaf322 (PMC12416565; doi:10.1093/braincomms/fcaf322)

**Supplementary Table 1 Variable means by sex and *APOE4* carrier status**

| **Characteristic** | **Women (*n* = 43)** | **Men (*n* = 25)** | ***APOE4* Carrier (*n* = 18)** | ***APOE4* Noncarrier (*n* = 50)** |
|---|---|---|---|---|
| Circadian rhythms (mean, SD) | | | | |
| Intradaily variability | 0.88 (0.17) | 0.97 (0.22) | 0.89 (0.17) | 0.92 (0.20) |
| Acrotime (hour of day) | 14.47 (1.69) | 13.89 (1.15) | 13.77 (1.51) | 14.43 (1.52) |
| Aβ PET SUVR (mean, SD) | 1.02 (0.19) | 0.96 (0.13) | 1.11 (0.27) | 0.96 (0.097) |
| Cognition (mean, SD) | | | | |
| Short delay free recall score | 10.3 (3.6) | 10.4 (3.1) | 11.5 (2.7) | 9.96 (3.5) |
| Digit Symbol Substitution score | 63.1 (15.4) | 57.0 (14.2) | 66.0 (12.2) | 59.0 (15.8) |
| Tau PET SUVR (*n* = 67) (mean, SD) | **(*n* = 42)** | **(*n* = 25)** | **(*n* = 18)** | **(*n* = 49)** |
| Braak region of interest I/II | 1.21 (0.13) | 1.18 (0.11) | 1.17 (0.12) | 1.21 (0.13) |
| Braak region of interest III/IV | 1.16 (0.10) | 1.13 (0.090) | 1.15 (0.15) | 1.14 (0.074) |

Related to Table 1**.** Positron Emission Tomography (PET); Standard Uptake Value Ratio (SUVR); California Verbal Learning Test (CVLT); Wechsler Adult Intelligence Scale (WAIS).

**Supplementary Figure 1. Distributions of circadian rhythms variables**, Related to Figure 1. Histograms for A) Acrotime (*n* = 67, 1 outlier removed) and B) Intradaily Variability (*n* = 68).

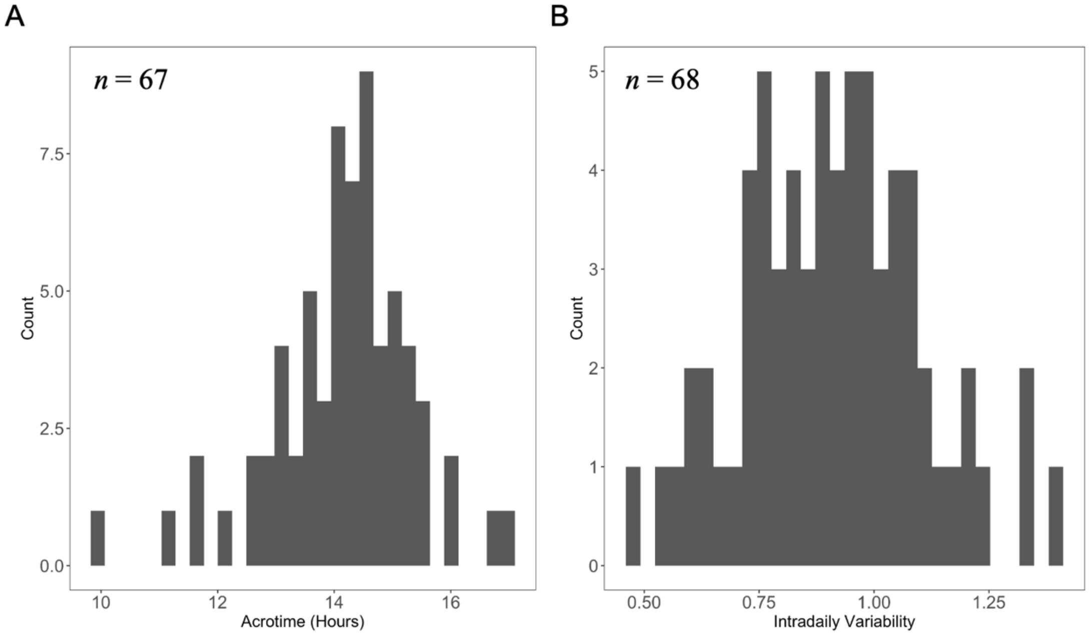

Supplement: fcaf322_Supplementary_Data [file fcaf322_supplementary_data.pdf]
